# Red Blood Cell Sedimentation Index Using Shear Stress of Blood Flow in Microfluidic Channel

**DOI:** 10.3390/bios12070547

**Published:** 2022-07-21

**Authors:** Yang Jun Kang

**Affiliations:** Department of Mechanical Engineering, Chosun University, 309 Pilmun-daero, Dong-gu, Gwangju 61452, Korea; yjkang2011@chosun.ac.kr; Tel.: +82-62-230-7052; Fax: +82-62-230-7055

**Keywords:** red blood cell sedimentation index, shear stress, microfluidic coflowing channel, erythrocyte sedimentation rate, blood syringe, microscopic image intensity

## Abstract

Red blood cell sedimentation has been used as a promising indicator of hematological diseases and disorders. However, to address several issues (i.e., syringe installation direction, blood on-off flow control, image-based quantification, and hemodilution) raised by the previous methods, it is necessary to devise a new method for the effective quantification of red blood cell sedimentation under a constant blood flow. In this study, the shear stress of a blood flow is estimated by analyzing an interface in a co-flowing channel to quantify the red blood cell sedimentation in blood syringes filled with blood (hematocrit = 50%). A red blood cell sedimentation index is newly suggested by analyzing the temporal variations in the shear stress. According to the experimental investigation, the sedimentation index tends to decrease at a higher flow rate. A higher level of hematocrit has a negative influence on the sedimentation index. As a performance demonstration of the present method, the red blood cell sedimentation processes of various test bloods were quantitatively compared in terms of the shear stress, image intensity, and sedimentation velocity. It was found that the proposed index provided a more than 10-fold increase in sensitivity over the previous method (i.e., image intensity). Additionally, it provided more consistent results than another conventional sedimentation method (sedimentation velocity). In conclusion, the present index can be effectively adopted to monitor the red blood cell sedimentation in a 10-min blood delivery.

## 1. Introduction

Red blood cells (RBCs) are thought to be the most common components of blood and are considered as extremely deformable. RBCs have substantial impacts on micro blood flows. Alterations of RBCs can be monitored physically by quantifying certain mechanical properties (RBC aggregation, RBC deformability, viscoelasticity, etc.) under a capillary blood flow [1,2,3]. Among the mechanical properties of blood, RBC aggregation occurs at a sufficiently low shear flow or stasis. In contrast, RBCs disaggregate at higher shear flow rates. RBC aggregation is highly dependent on aspects such as the membrane viscoelasticity, surface charge, and plasma protein [4,5]. As RBC aggregation provides quantitative information on the individual or interaction effects of plasma and RBCs [6], it has been regarded as a promising indicator for detecting infections, cardiovascular diseases, metabolic disorders, and hematological diseases [7,8,9]. The erythrocyte sedimentation rate (ESR), as calculated using the Westergren technique, is considered as a simple and gold standard for determining RBC aggregation. Without an external actuator, RBCs are sedimented owing to the gravitational force in a tube. According to this measurement method, the ESR is obtained as a sedimentation velocity (mm/h) from a visual detection of the sedimentation front with an elapse of 1 h. During the sedimentation of the RBCs, they aggregate and form continuous networks [10]. The networks of the colloidal gels then collapse [11]. 

Several techniques have been applied to quantify RBC aggregation or the ESR, including those based on electrical impedance [5,12,13,14,15], optical light intensity [7,16,17], microscopic image intensity [18,19], interface detection in the tube [11,20,21], a holographic laser tweezer [6], and the shear stress in microfluidic device [22]. Additionally, an external mechanism (e.g., a pinch valve [16], vibration motor [7], driving syringe [20], or vacuum pump [22]) can be adopted to periodically run or stop the blood flow in a microfluidic channel. Ultrasonic transducers have been suggested as an option for accelerating the RBC sedimentation in reservoirs [23]. Previous methods have measured the RBC aggregation or ESR in dextran-induced blood [24] or clinical disease blood [7,19,20,25]. More recently, our group suggested simple methods for quantifying the ESR using a microfluidic platform. By controlling a driving syringe filled with blood in a periodic on-off fashion, the ESR and RBC aggregation were obtained by analyzing the microscopic image intensity [26]. Notably, when blood is supplied into a microfluidic device from a driving syringe that has been installed horizontally, the hematocrit (Hct) of the RBCs tends to decrease over time. After a certain amount of time, the diluent is separated from the blood in the syringe. The blood and diluent are supplied sequentially into the microfluidic device. The contributions of the Hct can then be quantified by measuring the viscosity, as well as the junction pressure [27]. However, conventional and/or modified ESR techniques adopt a vertical installation of a driving syringe or tube [19]. During the RBC sedimentation in the syringe, the RBCs become concentrated at the bottom position, and diluted at the sedimentation front [28]. When blood is suppled into the microfluidic channel from the blood syringe, the Hct tends to increase over time. Additionally, when quantifying the ESR using the microscopic image intensity, it is necessary to periodically turn the syringe pump on and off. Lastly, as RBC sedimentation increases at lower levels of Hct, diluted blood (i.e., Hct = 25%) is generally prepared to quantify the ESR within a short time [27,29]. In view of the several issues raised by the previous studies (i.e., syringe installation direction, on-off blood flow, image intensity, blood dilution), it is necessary to devise a new and simple method for quantifying RBC sedimentation under a constant micro blood flow. 

In this study, by referring to the threshold shear stress for aggregating RBCs under a blood flow [22,30], the shear stress in microfluidic channel is suggested as an index for quantifying the RBC sedimentation in the blood syringe. The shear stress represents fluidic resistance of blood flow under continuous blood flow. It is determined by blood viscosity as well as blood flow rate. The shear stress could be considered as better effective than image intensity of blood flow. Thus, the shear stress is newly suggested to quantify red blood cell sedimentation under continuous blood flow. In this case, the Hct is set to a normal range (i.e., Hct = 50%). A driving syringe filled with blood is installed vertically. The RBC sedimentation in the syringe contributes to increasing the Hct of the blood supplied into the microfluidic channel over time. The shear stress of the blood flow is estimated by analyzing an interface in a coflowing channel partially filled with blood and a reference fluid. Based on the temporal variations of shear stress, a new index is then suggested for RBC sedimentation. The index can be used to estimate the RBC sedimentation in a blood syringe effectively. 

In contrast to the previous studies, the present method does not require stopping or restarting the blood flow (i.e., it works with a continuous blood flow). To provide settings similar to those of the conventional ESR technique, a driving syringe is installed vertically (i.e., vertical installation). Instead of the microscopic image intensity, the shear stress is used to obtain the RBC sedimentation index, especially under continuous blood flow. The present study does not require hemodilution to increase the sensitivity. In particular, the proposed index provides a more than 10-fold increase in sensitivity over the previous method (i.e., image intensity).

## 2. Materials and Methods

### 2.1. Microfluidic-Based Experimental Setup

**Figure 1:**
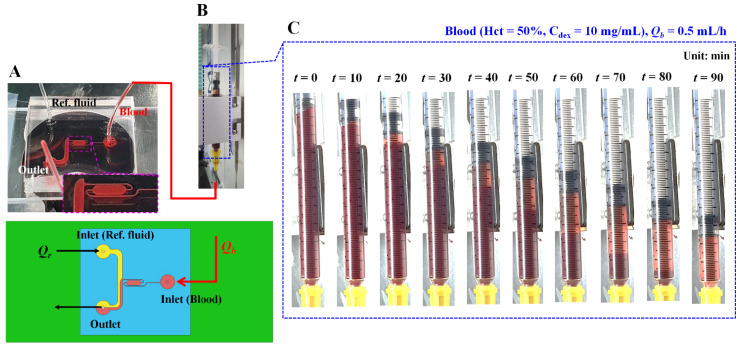
Microfluidic platform for quantifying red blood cell (RBC) sedimentation in terms of previous suggested factors. (**A**) Schematics of a microfluidic device (i.e., prototype, and 3D model). Microfluidic device with two inlets, outlet, two guiding channels (reference fluid channel and blood channel), and coflowing channel. Lower-side panel shows blood channel filled with blood. (**B**) Syringe pump for delivering blood as well as reference fluid. *Q_r_* and *Q_b_* denote flow rates of reference fluid and blood sample, respectively. (**C**) Snapshots for showing RBC sedimentation in the blood syringe for 90 min. In this case, blood (Hct = 50%) was prepared by mixing normal RBCs into dextran solution (10 mg/mL). Flow rate of syringe pump set to *Q_b_* = 0.5 mL/h.

As shown in Figure 1A, microfluidic device reported in a previous study [27] was reused to quantify the ESR in a driving syringe. The microfluidic device was designed with two inlets for two fluids (i.e., blood and reference fluid), two guiding channels (blood channel and reference fluid channel), a coflowing channel (width = 1000 µm, length = 3500 µm) filled with the blood and reference fluid, and an outlet. The lower panel of Figure 1A shows 3D CAD model of a microfluidic device. The depth of all channels was fixed at 50 µm. A polydimethylsiloxane (Sylgard 184, Dow Corning, Midland, MI, USA) device was fabricated using a soft lithography technique. The device was placed on an inverted optical microscope (IX53, Olympus, Tokyo, Japan) equipped with a 4× objective lens (numerical aperture = 0.1). One Tygon tubing (Cole-Parmer, Vernon Hills, IL, USA, ID = 0.01 inch, OD = 0.03 inch, and length = 300 mm) was inserted into each inlet. Another Tygon tubing (length = 200 mm) was fitted to the outlet. To repel air in the device and tubing, 1× phosphate-buffered saline (PBS) was injected through the tubing connected to the outlet. A reference syringe was filled by adding a glycerin solution (30%) to a disposable syringe (~1 mL). Simultaneously, a blood syringe was filled by adding blood into a disposable syringe. Thereafter, as depictured in Figure 1B, both syringes were installed into a syringe pump (neMESYS, Cetoni GmbH, Korbußen, Germany) aligned along the gravitational direction. The flow rates of each individual syringe (*Q_r_*: flow rate of reference fluid, *Q_b_*: flow rate of blood) were set to constant values. As a preliminary study, to understand the contribution of the RBC sedimentation in the blood syringe to the blood flow in the microfluidic channel, the RBC sedimentation was monitored by quantifying the sedimentation front in the blood syringe. The blood (Hct = 50%) was prepared by adding normal RBCs to a dextran solution (10 mg/mL). The flow rate of the blood syringe was set to 0.5 mL/h. Figure 1C shows snapshots of the RBC sedimentation in the blood syringe over 90 min. As blood was supplied to the microfluidic device from the blood syringe, the blood volume (or height) in the blood syringe decreased linearly over time. The RBC sedimentation contributed to the gradual increase in the diluent volume (or height) over time. 

### 2.2. RBC Sedimentation Index Using Temporal Variation of Shear Stress

**Figure 2:**
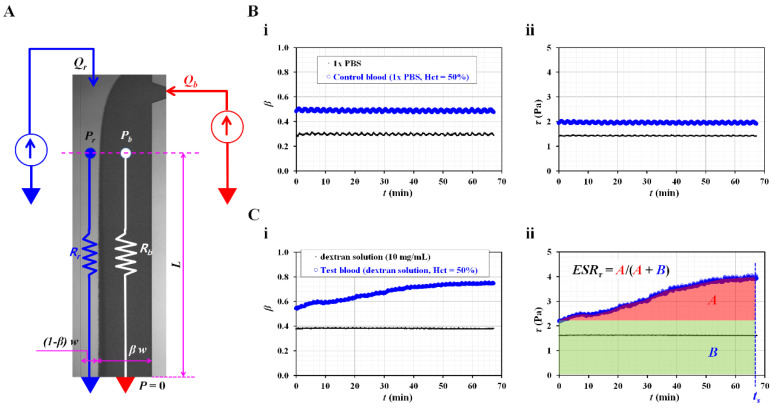
RBC sedimentation index in terms of shear stress of blood flow. (**A**) Discrete fluidic circuit model for estimating shear stress of blood flow in coflowing channel. (**B**) Variations of shear stress for control blood with no RBC sedimentation. In this case, control blood (hematocrit = 50%) was prepared by adding normal RBCs into 1× phosphate-buffered solution (PBS). (**i**) Temporal variations of interface (*β*) with respect to 1× PBS as well as control blood. (**ii**) Temporal variations of shear stress with respect to 1× PBS as well as control blood. (**C**) Variations of shear stress for sedimentation-enhanced test blood. Test blood (hematocrit = 50%) was prepared by adding normal RBCs into dextran solution (10 mg/mL). (**i**) Temporal variations of interface (*β*) with respect to dextran solution and test blood. (**ii**) Temporal variations of shear stress with respect to dextran solution and test blood. From the results, RBC sedimentation index was suggested as *ESR_τ_* = *A*/(*A* + *B*). Based on the temporal variations of *τ*, *A* and *B* were calculated as *A* = $\int_{0}^{t_{s}}\left(\tau \left(t\right)-\tau \left(t=0\right)\right)dt$ and *B* = $\int_{0}^{t_{s}}\tau \left(t=0\right)dt$.

Instead of the image intensity of the blood flow in the microfluidic channel, the shear stress was suggested as an index for quantifying the RBC sedimentation in the blood syringe. As shown in Figure 2A, the co-flowing channel was modeled with discrete fluidic circuit elements, such as the fluidic resistance and flow rate. A single co-flowing channel was partially filled with the reference fluid and blood. The width of each fluid was given as (1 − *β*) × *w* and *β* × *w*. According to previous studies [31,32,33,34], the virtual wall concept was used to simplify complex problems. In particular, the co-flowing channel was assumed to be two independent channels (i.e., a reference fluid channel and blood channel) connected in parallel. A correction factor (*C_P_*), which was expressed as interface (*β*), was then suggested to compensate for the mathematical modeling error resulting from the difference between the real physical model and simple mathematical model [35,36]. The frictional losses of the fluids were represented by two fluidic resistances (*R_r_* and *R_b_*), where the subscripts *r* and *b* represented the reference fluid and the blood, respectively. The flow rates of the two fluids were denoted by *Q_r_* and *Q_b_*, respectively. A symbol (▽) represented the zero value of the gauge pressure (i.e., ground, *p* = 0). At the distance of *L* from the ground, the pressure of each fluid was designated as *P_r_* or *P_b_*, respectively. In this case, both pressures had the same values in the straight and rectangular channels (i.e., *P_r_* ≈ *P_b_*). The pressure of each fluid was then derived as [35],

(1) $$P_{r}=\frac{12\;\mu_{r}\times L\times Q_{r}}{\left(1-\beta \right)\times w\times h^{3}}$$

In addition,

(2) $$P_{b}=\frac{12\;\mu_{b}\times L\times Q_{b}}{C_{P}\times \beta \times w\times h^{3}}$$

In Equations (1) and (2), *µ_r_* and *µ_b_* denoted the viscosities of the reference fluid and blood, respectively. *L* represented channel length of coflowing channel. Based on the same pressure condition, the blood viscosity formula could be derived as,

(3) $$\mu_{b}=\mu_{r}\times \left(\frac{\beta }{1-\beta }\right)\times \left(\frac{Q_{r}}{Q_{b}}\right)\times C_{P}\left(\beta \right)$$

According to the force balance between the pressure-induced force and viscous shear force along reference fluid stream [35], the relationship between shear stress and pressure difference was given as,

(4) $$\tau_{r}\times \left(2\left(1-\beta \;\right)\;w\;L\right)=P_{r}\times \left(\left(1-\beta \;\right)\;w\;h\right)$$

Similarly, the relationship between shear stress and pressure difference along blood stream was expressed as,

(5) $$\tau_{b}\times \left(2\;\beta \;w\;L\right)=P_{b}\times \left(\beta \;w\;h\right)$$

By substituting Equations (4) and (5) into Equations (1) and (2), the shear stress of each fluid stream was then derived as,

(6) $$\tau_{r}=\frac{6\;\mu_{r}\times Q_{r}}{\left(1-\beta \right)\times w\times h^{2}}$$

In addition,

(7) $$\tau_{b}=\frac{6\;\mu_{b}\times Q_{b}}{C_{P}\times \beta \times w\times h^{2}}$$

By substituting Equation (3) into Equation (7), both fluid streams satisfied with the same shear stress condition (i.e., *τ_r_* = *τ_b_ = τ*). According to Equations (3) and (6), the blood viscosity and shear stress of blood stream could be quantified by monitoring the interface (*β*) at a specific flow rate of the two fluids. For a rectangular microfluidic channel (i.e., width = 1000 µm, depth = 50 µm), the correction factor was given as *C_P_* = −9.014 *β*^4^ + 21.273 *β*^3^ − 18.403 *β*^2^ + 7.051 *β* − 0.168 (*R*^2^ = 0.99) [27]. According to Equations (1) and (6), pressure of reference fluid (*P_r_*) was expressed as *P_r_* = *τ_r_* × *L*/*h*. Namely, pressure was proportional to shear stress. As *L*/*h* was fixed in the identical microfluidic channel, the accuracy of shear stress formula was the same as the accuracy of pressure formula. Based on the previous work [27], normalized difference between analytical formula and numerical simulation was less than 6%. Thus, the Equation (6) could be used to monitor change in shear stress in the coflowing channel with enough accuracy.

To validate the contribution of the RBC sedimentation to the shear stress of the blood flow in the microfluidic channel, control blood (Hct = 50%) was prepared by adding normal RBCs to 1× PBS. As the diluent (1× PBS) did not include plasma proteins and the Hct was set to a higher level of 50%, the variations in the RBC sedimentation could be negligible within a short duration of 2 h [19]. The flow rate of both fluids was set to 0.5 mL/h. As shown in Figure 2(Bi), temporal variations of the interface (*β*) were identified with respect to the pure diluent (1× PBS) and control blood. As expected, neither fluid contributed to the varying *β* over time. Based on Equation (6), the shear stresses of both fluids were calculated over time. As shown in Figure 2(Bii), the temporal variations in shear stress were obtained with respect to the 1× PBS and control blood. The shear stresses of both fluids remained unchanged over time (i.e., shear stress of 1× PBS = 1.42 ± 0.01 Pa, shear stress of control blood = 1.95 ± 0.03 Pa). Then, to enhance the RBC aggregation in the control blood, the 1× PBS was replaced with a dextran solution (10 mg/mL). Test blood (Hct = 50%) was prepared by adding normal RBCs to the dextran solution. As shown in Figure 2(Ci), temporal variations in *β* were identified with respect to the pure dextran solution and test blood. As the pure dextran solution did not include RBCs, it did not contribute to the change in *β* over time. However, the test blood exhibited variations of *β* over time. Namely, the pure dextran solution influenced the RBC aggregation in the test blood. Figure 2(Cii) shows the temporal variations in *τ* with respect to the pure dextran solution and test blood. In this case, at an initial time (*t* = 0), the corresponding shear stresses of each fluid were obtained as *τ* = 1.61 ± 0.01 Pa (pure dextran solution) and *τ* = 2.18 ± 0.01 Pa (test blood), respectively. The shear stress of the test fluid increased with time. Referring to an RBC aggregation index reported in a previous study [7,37], the RBC sedimentation index was newly suggested as *ESR_τ_* = *A*/(*A* + *B*). Based on the temporal variations in *τ*, *A* and *B* were calculated as *A* =$\;\int_{0}^{t_{s}}\left(\tau \left(t\right)-\tau \left(t=0\right)\right)dt$ and *B* = $\int_{0}^{t_{s}}\tau \left(t=0\right)dt$. For the control blood (Figure 2(Bii)), *ESR_τ_* was calculated as zero, because A was zero. However, the RBC sedimentation index of the test blood was estimated as *ESR_τ_* = 0.30 ± 0.02 (*n* = 3). 

From the preliminary results, the RBC sedimentation index (ESR_τ_) showed substantial differences between the control and test blood.

### 2.3. Quantification of Flow Rate of Reference Fluid with Micro Particle Image Velocimetry

**Figure A1:**
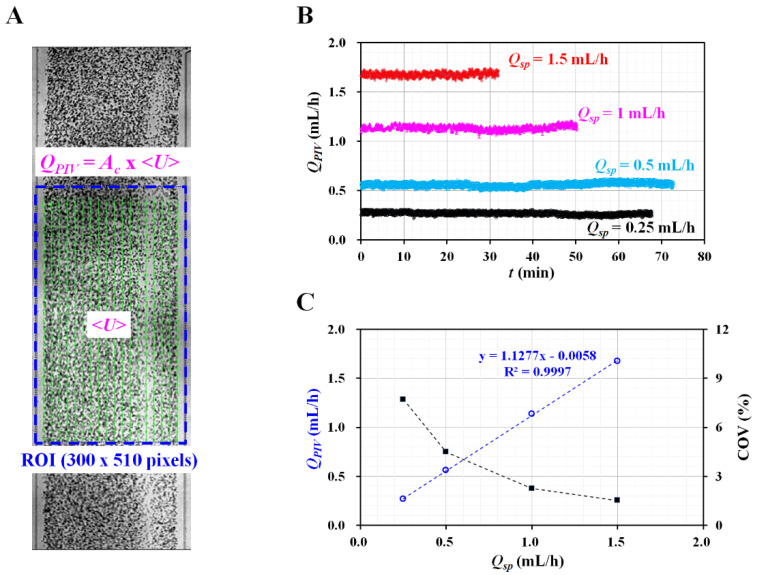
Validation of flow rate of reference fluid controlled by syringe pump. (**A**) Quantification of flow rate of reference fluid with microparticle image velocimetry. (**B**) Temporal variations of *Q_PIV_* with respect to flow rate of syringe pump (*Q_sp_*) = 0.25, 0.5, 1, and 1.5 mL/h. (**C**) Variations of *Q_PIV_* and coefficient of variance (COV) with respect to *Q_sp_*.

To visualize the velocity fields of the reference fluid, RBCs (20 µL) were added to the reference fluid (1 mL). A high-speed camera (FASTCAM MINI, Photron, Tokyo, Japan) was used to capture microscopic blood flow images at intervals of 1 s. The frame rate of the high-speed camera was set to 2000 frames/s. Two sequential images were continuously captured at intervals of 2 s. As shown in Figure A1A, a region of interest (ROI) of 300 × 510 pixels was selected in the straight channel. The velocity fields of the reference fluid were obtained by setting the integration size to 32 × 16 pixels (i.e., a 50% overlap between two sequential images) [38]. The average velocity (<*U*>) was then obtained by averaging the velocity fields distributed over the ROI. The flow rate of the reference fluid was calculated using *Q_PIV_* = *A_c_* × <*U*>. In this case, *A_c_* denoted the cross-sectional area of the rectangular channel (*A_c_* = width × depth).

### 2.4. Blood Preparation for Stimulating RBC Sedimentation in the Driving Syringe

An RBC bag (~320 mL) filled with concentrated RBCs was provided by the Gwangju–Chonnam Blood Bank (Gwangju, South Korea). It was stored in a refrigerator at 4°. According to the specific washing procedures [23], normal RBCs were then collected from the concentrated RBCs. To enhance the aggregation within the normal RBCs, normal RBCs were added to the dextran solution. Nine dextran solutions (C_dex_ = 5, 7.5, 10, 15, 20, 30, 40, 60, and 80 mg/mL) were prepared by dissolving dextran powder (*Leuconostoc* spp., MW = 450–650 kDa; Sigma-Aldrich, St. Louis, MO, USA) in 1× PBS. Unless otherwise stated, the Hct was set at 50%.

## 3. Results and Discussion

### 3.1. Red Blood Cell (RBC) Sedimentation in Driving Blood Syringe and Its Contribution to Blood Flow

**Figure 3:**
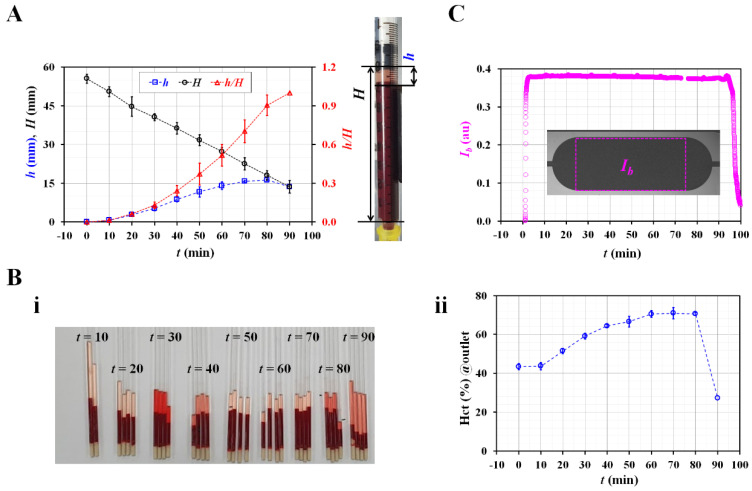
Quantification of RBC sedimentation in terms of three representative factors (i.e., sedimentation distance, hematocrit, and image intensity). (**A**) Temporal variations of RBC sedimentation distance in blood syringe. Right-side panel shows diluent height (*h*) as well as blood height (*H*). (**B**). Measurement of hematocrit with hemocytometer. (**i**) Capillary tubes captured after operation of micro hemocytometer. (**ii**) Temporal variations of hematocrit of blood collected at outlet. (**C**) Temporal variation of image intensity (*I_b_*) in blood channel.

Three representative factors (diluent volume/blood volume, Hct, and image intensity) were used to quantify the effects of the RBC sedimentation in the blood syringe. First, based on the snapshots (Figure 1C), the diluent height (*h*) and blood height (*H*) were obtained by inspecting the sedimentation front in the blood syringe over time. Figure 3A shows the temporal variations in *h*, *H*, and *h*/*H*. *h* gradually increased for up to 80 min. The right panel shows the diluent height (*h*) and blood height (*H*). *H* gradually decreased over time. The RBC sedimentation caused an increase in *h*/*H* ranging from 0 to 1. The RBC sedimentation caused a decrease in the Hct of the blood in the blood syringe. As the total blood volume was fixed in the blood syringe, it was expected that the Hct of the blood supplied from the blood syringe would increase over time. Second, to measure the Hct of the blood in the microfluidic channel, blood was collected from the outlet at intervals of 10 min. Thereafter, the variations in Hct were identified using a micro hemocytometer (Microhematocrit, VS-12000, Vison Scientific Co., Daejeon, South Korea). Figure 3(Bi) shows an image of the capillary tubes captured after operation of the micro hemocytometer from *t* = 10 min to *t* = 90 min. As shown in Figure 3(Bii), the Hct values were obtained at intervals of 10 min. As expected, the Hct increased gradually from *t* = 10 min to *t* = 80 min. The Hct values at specific times were measured as Hct = 43.7 ± 1.5% (*t* = 10 min), 51.5 ± 2% (*t* = 20 min), 64.4 ± 1.6% (*t* = 40 min), 70.5 ± 2.8% (*t* = 60 min), 70.6 ± 2.8% (*t* = 80 min), and 27.4 ± 0.8% (*t* = 90 min). This indicated that the RBC sedimentation contributed to a continuous increase in the Hct of the blood in the microfluidic channel. Finally, the image intensity of the blood flow (*I_b_*) was obtained by analyzing the image intensity of the blood flows within the blood channel (Region-Of-Interest, 2 × 1 mm^2^). Figure 3C shows the temporal variations in *I_b_* over time. *I_b_* remained unchanged over time. From these results, it was considered as impossible to monitor the RBC sedimentation in terms of the image intensity of the blood flow, especially under a continuous blood flow. Thus, it was inferred that the RBC aggregation [14] should be measured after stopping the blood flow [7,16]. 

### 3.2. Contributions of Flow Rate and Hematocrit to RBC Sedimentation Index

As shown in Figure 1C, it was expected that the RBC sedimentation in the blood syringe could vary according to the flow rate and Hct. Thus, it was necessary to evaluate the contributions of these two representative factors to the RBC sedimentation index. 

According to Equation (6), to obtain a consistent value of the shear stress, the flow rate of the reference fluid should remain consistent over a sufficient period. The fluctuations in the flow rate controlled by the syringe pump were validated using microparticle image velocimetry. The flow rate of the syringe pump varied from *Q_sp_* = 0.25 mL/h to *Q_sp_* = 1.5 mL/h. Figure A1B shows the temporal variations in *Q_PIV_* with respect to *Q_sp_.* As a result, it can be seen that the flow rate of the reference fluid remained constant over time. To quantify the fluctuations of the flow rate, the coefficient of variance (COV) was calculated as COV = standard deviation/mean. Figure A1C shows the variations of *Q_PIV_* and the COV with respect to setting the flow rate of the syringe pump (*Q_sp_*). A linear regression analysis gives a higher value of the regression coefficient (i.e., *R*^2^ = 0.9997). By increasing the flow rate of the syringe pump from 0.25 mL/h to 1.5 mL/h, the COV decreased substantially, from 7.7% to 1.5%. At a flow rate of *Q_sp_* = 0.5 mL/h, the flow rate of the reference fluid was measured as *Q_PIV_* = 0.56 ± 0.03 mL/h. The COV was then calculated as 4.5%. These results indicate that syringe pump can consistently maintain the flow rate of the reference fluid for a long period of 70 min.

**Figure 4:**
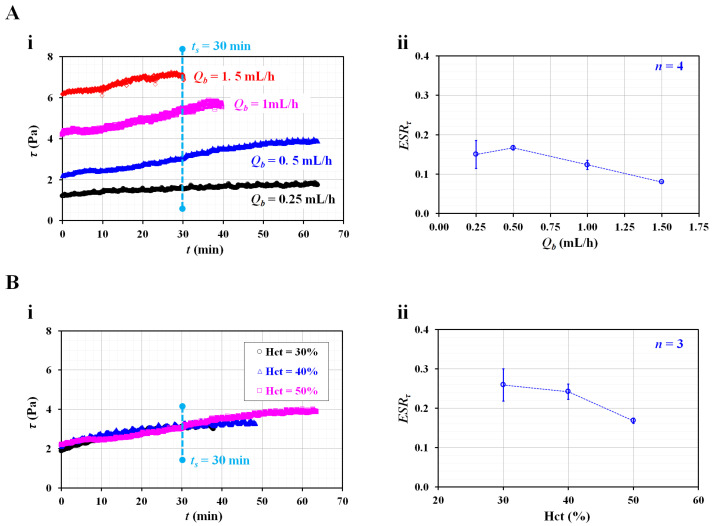
Contributions of flow rate and hematocrit to RBC sedimentation index (*ESR_τ_*). (**A**) Effect of blood flow rate on RBC sedimentation index. (**i**) Temporal variation of shear stress with respect to blood flow rate (*Q_b_*). (**ii**) Variation of *ESR_τ_* with respect to *Q_b_*. (**B**) Effect of hematocrit on RBC sedimentation index. (**i**) Temporal variation of shear stress with respect to Hct = 30%, 40%, and 50%. (**ii**) Variation of *ESR_τ_* with respect to Hct.

Next, the contribution of the flow rate the RBC sedimentation index (ESR_τ_) was validated by varying the flow rates from 0.25 mL/h to 1.5 mL/h. The flow rates of both fluids were set to the same value (i.e., *Q_r_* = *Q_b_*). The test blood (Hct = 50%) was prepared by adding normal RBCs to a dextran solution (10 mg/mL). Figure 4(Ai) shows the temporal variations in the shear stress (*τ*) with respect to the blood flow rate (*Q_b_*). At *t* = 0, the shear stress (*τ*) increases substantially at higher flow rates. A higher flow rate causes a decrease in the delivery time. Based on the delivery time of *Q_b_* =1.5 mL/h, the integration time for calculating *A* and *B* was limited to 30 min (i.e., *t_s_* = 30 min). Figure 4(Aii) shows the variations in the RBC sedimentation index (ESR_τ_) with *Q_b_*. The ESR_τ_ is represented as the mean ± standard deviation (*n* = 4). The sedimentation index does not exhibit a substantial difference between *Q_b_* = 0.25 and *Q_b_* = 0.5 mL/h. In addition, it includes large fluctuations at *Q_b_* = 0.25 mL/h. Above *Q_b_* = 0.5 mL/h, the sedimentation index tends to decrease at higher flow rates. Namely, when the syringe pump is set to a higher flow rate, the RBC sedimentation decreases. Therefore, the RBC sedimentation index decreases. The results indicate that the flow rate of the syringe pump has a strong influence on the RBC sedimentation index. Thus, the flow rates of both fluids were fixed as *Q_r_* = *Q_b_* = 0.5 mL/h for the following experiments. 

According to previous studies [13,19,20,27], a higher Hct level could retard RBC sedimentation in the tube or syringe. To evaluate the contribution of the Hct to the RBC sedimentation index, the sedimentation index was measured with respect to Hct = 30%, 40%, and 50%. Figure 4(Bi) shows the temporal variations in the shear stress with respect to the Hct. Using the temporal variations in *τ*, the RBC sedimentation index was obtained with respect to Hct. The integration time was fixed at *t_s_* = 30 min. As shown in Figure 4(Bii), the ESR_τ_ gradually decreases with respect to Hct. The sedimentation index is expressed as the mean ± standard deviation (*n* = 3). As expected, a higher Hct level negatively affects RBC sedimentation. Compared with previous studies [7,20,24], the present results exhibited sufficiently reliable trends with respect to Hct. From the results, and for obtaining a consistent RBC sedimentation index, the Hct of test fluid was fixed at 50% for the following experiments. 

### 3.3. Quantitative Validation of Suggested RBC Sedimentation Index

As the last demonstration, it was necessary to validate the suggested RBC sedimentation index by comparing it with previous methods (i.e., sedimentation height per 1 h and image intensity). A quantitative comparison between the present method and previous methods was conducted using RBC sedimentation-enhanced blood. Based on previous studies [39,40,41], the dextran solution contributed to increasing the RBC aggregation or sedimentation.

**Figure 5:**
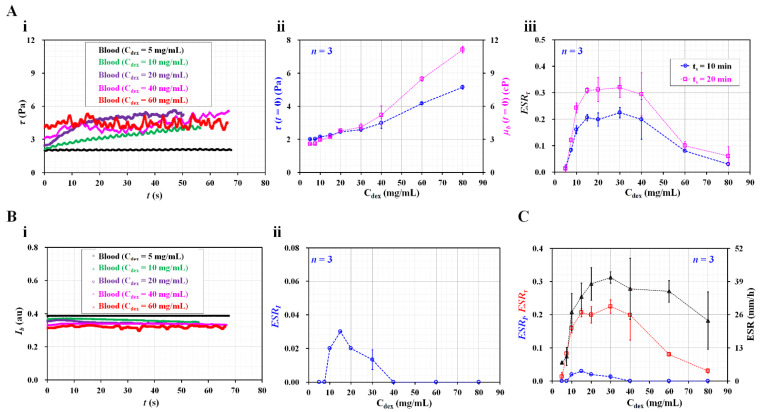
Quantitative comparison between present method and previous methods. (**A**) Contribution of dextran solution to RBC sedimentation index. (**i**) Temporal variations of shear stress with respect to C_dex_. (**ii**) Variations of initial shear stress (τ (*t* = 0)) as well as blood viscosity (*µ_b_* (*t* = 0)) with respect to C_dex_. (**iii**) Variations of RBC sedimentation index with respect to C_dex_ and *t_s_*. (**B**) Evaluation of RBC sedimentation using image intensity of blood flow (*I_b_*). (**i**) Temporal variations of *I_b_* with respect to C_dex_. (**ii**) Variations of ESR_I_ with respect to C_dex_. (**C**) Quantitative comparison of RBC sedimentation index in terms of shear stress, image intensity, and sedimentation velocity.

As shown in Figure 5(Ai), the temporal variations in τ were identified by increasing the concentrations of the dextran solution. The initial shear stress *τ* (*t* = 0) increases substantially at higher dextran solution concentrations. For two test blood samples (C_dex_ = 10 and 20 mg/mL), the shear stress tends to increase significantly over time. However, above C_dex_ = 40 mg/mL, the test blood exhibits fluctuations in the shear stress over time, rather than a substantial increase over time. To determine the effect of the pure diluent, the viscosity of the test blood and initial shear stress were obtained with respect to C_dex_. 

As shown in Figure 5(Aii), the variations in *τ* (*t* = 0) and *µ_b_* (*t* = 0) are represented with respect to C_dex_. At *t* =0, the blood viscosity and shear stress increase at higher dextran solution concentrations. From the results, it can be seen that the dextran solution contributes significantly to increasing the blood viscosity, as well as the shear stress. According to Equation (7), the shear stress is proportional to the blood viscosity. Thus, these results can be considered as sufficiently reliable. Based on the temporal variations in *τ*, the RBC sedimentation index (ESR_τ_) was obtained with respect to C_dex_. Furthermore, the test blood was saturated for 20 min (C_dex_ = 20 mg/mL). In this case, the integration time was selected as *t_s_* = 10 or 20 min. Figure 5(Aiii) shows the variations in the RBC sedimentation index with respect to C_dex_ and *t_s_*. The sedimentation index increases up to a C_dex_ of 15 mg/mL. It remains constant from C_dex_ = 15 mg/mL to C_dex_ = 40 mg/mL. The sedimentation index gradually decreases above C_dex_ = 40 mg/mL. Based on the peak value of the sedimentation index, the longer integration time (*t_s_* = 20 min) causes a 50% increase in sensitivity relative to the shorter integration time (*t_s_* = 10 min). 

In previous studies [7,20,23,37], the RBC aggregation or sedimentation was quantified by analyzing the image intensity of the blood flow over time. Following such previous methods, as shown in Figure 3C, the image intensity of the blood flow (*I_b_*) was obtained under a continuous blood flow (*Q_b_* = 0.5 mL/h). Figure 5(Bi) shows the temporal variations in *I_b_* with respect to C_dex_. The image intensity is higher at lower concentrations of dextran solution. The time variation of *I_b_* is not distinct with respect to C_dex_. Based on the definition of the RBC aggregation index [37], the ESR index (ESI_I_) was calculated using the temporal variations in *I_b_*. Figure 5(Bii) shows the variations in ESR_I_ with respect to C_dex_. The ESR_I_ exhibits a peak value of 0.03 at C_dex_ = 10 mg/mL. The ESR_I_ has a value of zero for C_dex_ = 40 mg/mL. As the variation in *I_b_* is much smaller over time, the ESR_I_ provides extremely low sensitivity within C_dex_ = 40 mg/mL. For this reason, the image intensity is ineffective for quantifying RBC sedimentation, especially under a continuous blood flow. In addition, a conventional ESR was quantified by monitoring the RBC sedimentation in disposable syringe. After injecting 1 mL of blood into the disposable syringe (~1 mL), the sedimentation front was monitored for 30 min. The conventional ESR was then obtained by dividing the sedimentation height at 0.5 h (i.e., sedimentation velocity, mm/h). Figure 5C shows a quantitative comparison of the RBC sedimentation index in terms of the shear stress (ESR_τ_), image intensity (ESR_I_), and sedimentation velocity (mm/h). Among the two data of the ESR_τ_ as shown in Figure 5(Aiii), the ESRτ calculated at a shorter integrating time (*t_s_* = 10 min) was redrawn with respect to C_dex_. The present sedimentation index exhibits very similar trends to those of the conventional ESR technique (i.e., sedimentation velocity). However, the ESR_τ_ shows more consistent trends than the sedimentation velocity. The sedimentation velocity exhibits a large scattering. Furthermore, for the test blood (C_dex_ = 20 mg/mL), the sensitivity of ESR_τ_ is more than 10-fold higher than that of ESR_I_. 

From the experimental investigation, it can be concluded that the RBC sedimentation index proposed in this study can be effectively used to quantify RBC sedimentation in a driving syringe. Furthermore, the sedimentation index yields consistent results when compared with the conventional ESR (sedimentation velocity). Thus, while supplying blood (Hct = 50%) into the microfluidic channel continuously for 10 min, it is possible to quantify RBC sedimentation effectively in terms of the shear stress, rather than the image intensity. One limitation of the present study is that the present index was not applied to test clinical blood. Furthermore, as the present method was demonstrated in a well-equipped laboratory, it will be necessary to update the present method for in-situ diagnoses.

## 4. Conclusions

In this study, to quantify the RBC sedimentation in blood syringes filled with blood (Hct = 50%), a new RBC sedimentation index was suggested, based on the shear stress of the blood flow in the microfluidic channel. Under a constant blood flow, the shear stress was estimated by analyzing an interface in a coflowing channel. According to an experimental investigation of the flow rate and Hct, the sedimentation index tended to decrease at higher flow rates. A higher Hct level had a negative effect on the RBC sedimentation index. As a performance demonstration, the RBC sedimentation values of various test blood samples were quantitatively compared in terms of the shear stress, image intensity, and sedimentation velocity. As a result, it was found that the proposed sedimentation index provides more than 10-fold higher sensitivity than the image intensity. It has the ability to provide more consistent results than the conventional ESR technique (sedimentation velocity). In the near future, the present sedimentation index will be applied to test clinical blood, and the method will be updated for in in-situ diagnosis.

## Data Availability

Not applicable.
